# Biopharmaceutical Understanding of Excipient Variability on Drug Apparent Solubility Based on Drug Physicochemical Properties: Case Study—Hypromellose (HPMC)

**DOI:** 10.1208/s12248-019-0411-1

**Published:** 2020-02-18

**Authors:** P. Zarmpi, T. Flanagan, E. Meehan, J. Mann, N. Fotaki

**Affiliations:** 1 Department of Pharmacy and Pharmacology, University of Bath, Claverton Down, Bath, BA2 7AY UK; 2 Pharmaceutical Technology & Development, AstraZeneca, Macclesfield, UK; 3 UCB Pharma, Chemin du Foriest, 1420 Braine-l’Alleud, Belgium

**Keywords:** excipient variability, HPMC, viscosity, drug solubility, physicochemical properties

## Abstract

Identification of the biopharmaceutical risks of excipients and excipient variability on oral drug performance can be beneficial for the development of robust oral drug formulations. The current study investigated the impact of Hypromellose (HPMC) presence and varying viscosity type, when used as a binder in immediate release formulations, on the apparent solubility of drugs with wide range of physicochemical properties (drug ionization, drug lipophilicity, drug aqueous solubility). The role of physiological conditions on the impact of excipients on drug apparent solubility was assessed with the use of pharmacopoeia (compendial) and biorelevant media. Presence of HPMC affected drug solubility according to the physicochemical properties of studied compounds. The possible combined effects of polymer adsorption (drug shielding effect) or the formation of a polymeric viscous layer around drug particles may have retarded drug dissolution leading to reduced apparent solubility of highly soluble and/or highly ionized compounds and were pronounced mainly at early time points. Increase in the apparent solubility of poorly soluble low ionized drugs containing a neutral amine group was observed which may relate to enhanced drug solubilization or reduced drug precipitation. The use of multivariate data analysis confirmed the importance of drug physicochemical properties on the impact of excipients on drug apparent solubility and revealed that changes in HPMC material properties or amount may not be critical for oral drug performance when HPMC is used as a binder. The construction of a roadmap combining drug, excipient, and medium characteristics allowed the identification of the cases where HPMC presence may present risks in oral drug performance and bioavailability.

**Electronic supplementary material:**

The online version of this article (10.1208/s12248-019-0411-1) contains supplementary material, which is available to authorized users.

## INTRODUCTION

The potential influence of excipients on the performance of solid oral dosage forms is a topic of great interest in terms of pharmaceutical Quality by Design (QbD). Further to their intended use, excipients may affect the properties and performance of final dosage forms leading to batch inconsistencies, altered bioavailability and bioinequivalence of products (1–3). Excipient variability (changes in material properties) and variation (changes in amount) constitute an additional obstacle to robust manufacturing, as changes in excipient material properties can undermine the critical quality attributes of the final product (4). Moreover, excipient inertness is questionable as their presence can influence oral drug absorption (5,6). Understanding the biopharmaceutical risks of excipient presence will pave the way for manufacturing products with robust product performance.

**Fig. 1:**
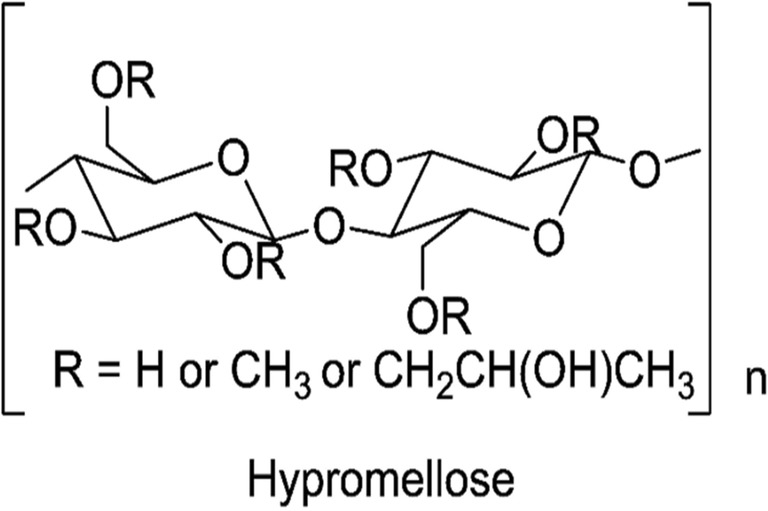
Chemical structure of Hypromellose (ChemDraw Professional 15.0)

Binders are typically used in solid dosage form manufacturing to promote adequate mechanical strength of granules or tablets. Hypromellose, also called hydroxypropyl methylcellulose (HPMC), is a polymeric binder used in wet granulation (7). HPMC is a water soluble non-ionic cellulosic polymer substituted with methoxy and hydroxypropyl groups (Fig. 1). In addition to its binding properties, HPMC is extensively used as a release controlling excipient. This dual functionality depends on excipient level (2% *w*/*w* to 5% *w*/*w* as binder and 10% *w*/*w* to 80% *w*/*w* as release modifier) and viscosity type (high viscosity grades are typically used to control drug release) (8). The effectiveness of HPMC can be influenced by its swelling and gelling properties which can delay drug dissolution or release (9,10) or by the presence of other excipient types in a formulation (11,12).

Molecular properties (molecular weight, degree of substitution, substitution pattern), particle properties (particle size distribution) and excipient level have been identified as critical material attributes affecting excipient and product performance (4). Molecular weight and excipient level directly relate to the formation of a viscous HPMC gel layer. HPMC brands of high molecular weight swell faster and form a thicker viscous layer compared to low molecular weight brands (9,13,14). The formation of thick viscous layers when increasing HPMC molecular weight is attributed to the slow rate of polymer erosion (9). Real-time surface dissolution UV imaging demonstrated the complex polymeric network formed by high molecular weight HPMC brands and the susceptibility of low molecular weight HPMC brands to erosion (15). Due to the proportionality of HPMC molecular weight and viscosity of its aqueous solutions, high molecular weight excipient brands correspond to high viscosity HPMC types. The formation and thickness of the gel layer depend also on the level of HPMC in solid dosage forms. Increasing the level of HPMC results in a concentrated and viscous gel layer either due to increased chain entanglement (16) or slow polymer erosion rate (17). A minimum excipient concentration (referred to as excipient percolation threshold) under which HPMC cannot form an effective viscous layer able to control drug release was reported (18). The percolation thresholds of different viscosity type HPMC brands were similar (20% *v*/*v* corresponding to 20% *w*/*w*) and showed consistent controlled drug release (verapamil HCl, United States Pharmacopeia (USP 2) apparatus, 50 rpm, 900 mL, phosphate buffer pH 7.5) from extended release tablets containing HPMC (18). Use of confocal laser scanning microscopy revealed that the early gel layer formation depends on the level of HPMC. Hydrophilic matrices containing 5% *w*/*w* and 10% *w*/*w* of HPMC formed a thin and heterogeneous gel layer initially (5–15 min), which could not be maintained due to the increased water uptake accelerating polymer erosion (19).

Several biopharmaceutical factors affecting the impact of HPMC on product performance have been identified. The performance of HPMC depends on the composition and properties of the dissolution medium. Salts (20), sugars (21) and food components (22) interact with the polymeric chains of HPMC and affect the formation of the gel layer. Moreover, bile salts were found to affect the thermal transition (gel formation) of cellulosic polymers. The hydrophobic parts of bile salts adsorb onto the hydrophobic regions of polymers and increase the excipient transition temperature upon heating (23,24). The pH of the dissolution medium can also affect the swelling/gelling properties of HPMC due to changes in the transport behaviour of water into the polymer, despite the non-ionic nature of HPMC. Rapid polymer hydration and larger excipient swelling were observed in media of basic (24% increase in polymer diameter after 200 min at pH = 6) compared to acidic pH (15% increase in polymer diameter after 200 min at pH = 2) [pH defined as basic and acidic according to the physiological pH range]. The authors highlighted the biopharmaceutical consequence of this polymeric response as faster drug release should be expected in the acidic gastric compared to the basic intestinal compartment (25).

The aim of this study was to assess the biopharmaceutical impact and criticality of HPMC variability and variation (when used as a binder in immediate release formulations) on drug apparent solubility. HPMC variability and variation were addressed by selecting three HPMC brands of different viscosity type using two different excipient levels (low: 2% *w*/*w*, high: 5% *w*/*w*). The biopharmaceutical implications of HPMC variability on drug apparent solubility were examined by choosing compounds of a wide range of physicochemical properties (drug ionization, drug lipophilicity, drug aqueous solubility) and media (compendial and biorelevant) simulating the gastrointestinal conditions. Multivariate data analysis (partial least squares (PLS)) and roadmaps were used to identify the critical role of certain variables (drug properties, excipient presence, medium characteristics) on the impact of HPMC on drug apparent solubility.

## MATERIALS AND METHODS

### Materials

APIs: sulfamethoxazole (SMX) and paracetamol (PRC) were obtained from Fisher Scientific (UK). Furosemide (FRS), itraconazole (ITZ) and dipyridamole (DPL) were obtained from VWR (UK). Ibuprofen (IBU), carbamazepine (CBZ) and metformin (MTF) were obtained from Fagron (UK). Excipients: HPMC-Sigma was obtained from Sigma Aldrich (UK). Pharmacoat 606 and Pharmacoat 615 were obtained from Shinetsu (Japan). Chemicals: acetic acid (> 99.7%), hydrochloric acid 36.5–38%, high-performance liquid chromatography (HPLC) grade methanol, HPLC grade acetonitrile, dichloromethane, pepsin (from porcine) were obtained from Sigma-Aldrich (UK). Maleic acid, sodium chloride, sodium hydroxide, potassium phosphate monobasic, sodium dihydrogen orthophosphate dihydrate, disodium hydrogen orthophosphate dihydrate, potassium dihydrogen orthophosphate, anhydrous sodium sulfate, HPLC grade trifluoroacetic acid were obtained from Fisher Scientific (UK). Sodium taurocholate (Prodotti Chimici Alimentari S.P.A., Italy), egg lecithin–Lipoid EPCS (Lipoid GmbH, Germany), were obtained from the sources specified. Water was ultra-pure (Milli-Q) laboratory grade. Filters: Whatman® 13 mm cellulose nitrate filters 0.45 μm pore size and polytetrafluoroethylene (PTFE) 13 mm filter 0.45 μm pore size were purchased from Fisher Scientific (UK).

### Instrumentation

Fisherbrand waterbath (Fisher Scientific, UK), Sartorius BP 210 D balance (Sartorius Ltd., UK), Buchi R114 Rotavapor (Buchi, Switzerland), Mettler Toledo SevenCompact S210 pH meter (Mettler Toledo, Switzerland), Vortex-Genie 2 vortex mixer (Scientific Industries Inc., USA), Agilent Technologies 1100 series HPLC system (quaternary pump (G1311A), autosampler (G1313A), thermostatted column compartment (G1316A), diode array detector (G1329A) and Chemstation software (Agilent Technologies, USA).

### Methods

#### Compounds Selected for Solubility Experiments

Compound selection was based on properties affecting oral drug absorption, namely ionization, lipophilicity and aqueous solubility (26). The compounds were chosen to cover a wide range of ionization (low ionized: F_(ion)_ < 50%, highly ionized: F_(ion)_ > 50%), drug lipophilicity (based on the drugs’ partition coefficient (log P): − 1.5 < log *P* < 6.5) and aqueous solubility (based on the compound’s BCS (Biopharmaceutical Classification System) classification (high: BCS class I and III; low: BCS class II and IV)) (27). The compounds used for the solubility experiments and their physicochemical properties (drug ionization, drug lipophilicity, drug aqueous solubility) are presented in Table I.

**Table I:** Physicochemical Properties and Structure of the Compounds Used for the Solubility Experiments (ChemDraw Professional 15)

	Drug	Ionization	Lipophilicity (log P)*	Solubility**
Neutral Drugs		Neutral (pKa=9.38) (28)	0.20 (28)	High (28)
		Neutral (pKa=15)^a^	2.45 (29)	Low (30)
Weak acids		Weak acid (pKa=3.8) (31)	2.29 (31)	Low (31)
		Weak acid (pKa=4.5) (32)	4.00 (33)	Low (32)
Weak Bases		Weak base (pKa=2.8) (34)	-1.43 (35)	High (34)
		Weak base (pKa=6.2) (36)	2.74 (37)	Low (38)
		Weak base (pKa=4.5) (39)	6.20 (39)	Low (40)
Ampholytes		Ampholyte [Weak base: pKa_1_=1.7/Weak acid:. pKa_2_=5.6] (41)	0.89^a^	Low (42)

*Experimental values, ** based on the compound’s BCS (Biopharmaceutical Classification System) classification (high: BCS Class I and III; low: BCS Class II and IV) (27), ^a^Source: DrugBank

#### Media Prepared for Solubility Experiments

Compendial media (0.1 N HCl pH 1, phosphate buffer pH 6.8) were prepared according to the method described in the European Pharmacopoeia (43). Fasted State Simulated Gastric Fluid (FaSSGF) and Fasted State Simulated Intestinal Fluid (FaSSIF-V2) were prepared as described by Jantratid *et al.* (44).

#### Design of Experiments Used for Solubility Experiments

A full-factorial design of experiments (DoE) was performed to determine the number of necessary experiments using StatGraphics Centurion XVII (Statpoint Technologies Inc., USA). As the composition of the studied media (pH, presence of bile salts) will affect drug solubility, two models for the DoE were constructed to discriminate between the effects of excipients on drug solubility in compendial (model 1) and biorelevant conditions (model 2). The examined factors were (i) compound (Table I), (ii) excipient brand (Pharmacoat 606, Pharmacoat 615, HPMC-Sigma), (iii) excipient level (low, high), (iv) medium (gastric, intestinal). The response was the impact of each excipient on drug solubility [expressed as the relative increase or decrease in presence compared to absence of excipient (section “Treatment of *In Vitro* Solubility Data”)]. A total of 96 × 3 experiments were determined for each model. A total of 16 × 3 additional experiments for each model were conducted to determine drug solubility in the corresponding media in the absence of excipient. These experiments were not included in the DoE as drug solubility in excipient absence was measured only for the calculation of relative excipient effects on drug apparent solubility.

#### Solubility Studies

Drug solubility studies in the absence and presence of excipient were performed in triplicate using the shake-flask method (45). Drug excess amount and 2% *w*/*w* or 5% *w*/*w* of each excipient were weighed and placed in centrifuge tubes. For poorly soluble drugs, the amount of excipient was determined considering an average of 500 mg tablet weight (46), which resulted in 9% *w*/*w* (10 mg of excipient and 100 mg of drug; low level) and 20% *w*/*w* (25 mg of excipient and 100 mg of drug; high level) of excipient in the total volume of the physical mixture. For highly soluble drugs, as higher drug excess amount was used to ensure saturation, the excipient amount was increased in order to keep the same % *w*/*w* of excipient in the total volume of the physical mixture as per the poorly soluble drugs. The physical mixtures were vortexed for 3 min. Five millilitres of each medium was added in the tubes and the samples were placed in a shaking water bath (37 °C, 200 strokes per minute (spm)). At 0.5, 4 and 24 h (for PRC, SMX, CBZ, DPL, IBU) and 24 h (for MTF, FRS, ITZ), 500 μL was sampled and filtered through PTFE filters (or cellulose nitrate filters for the cases of IBU and CBZ). Filter adsorption studies were prior performed in triplicate for each drug. No adsorption issues onto the filters used were observed for the studied drugs. Filtered samples were further diluted (if needed) with the corresponding medium and analysed by HPLC (Supplementary Table I). Analytical procedures for drug quantification in the samples were modifications of already published methods. Drug quantification was made based on calibration curves. Standards were formulated from concentrated stock solutions consisting of drug dissolved in MeOH. As drug solubility can be influenced by the changes in the pH of solutions by presence of dissolved drug (38), the pH of samples after the completion of each experiment was measured. Drug solubility was calculated based on the sample drug concentration measured. For neutral drugs, weak acids in acidic media and weak bases in basic media, the experimental drug solubilities determined the intrinsic solubility values. For weak acids and weak bases in basic and acidic media, respectively, the experimental drug solubilities determined drug solubility of the ionized molecules. The drug solubility measured was considered as the apparent drug solubility (dynamic solubility), as experimental points over a period of time were not available for the whole set of drugs to ensure that equilibrium solubility has been reached in 24 h for all the studied compounds.

#### Treatment of *In Vitro* Solubility Data

The relative effect (RE) of each excipient on drug apparent solubility was calculated based on Eq. 1:

1 $$ RE=\frac{\left(S- Sr\right)}{Sr}\times 100 $$

where *S* and *Sr* denote the drug solubility in presence and absence (reference solubility) of excipient at 0.5, 4 and 24 h. REs of excipients on drug solubility > 25% or < −20% were considered as significant change in drug apparent solubility to assess excipient criticality (this range was selected as a similar range is set in order to assess differences in drug exposure after oral administration, i.e. in bioequivalence studies) (47).

Box plots depicting the impact of excipients on drug solubility at 24 h for all the studied compounds or as a function of time (0.5, 4 and 24 h) for CBZ and DPL were constructed using Spotfire 7.10.1 (TIBCO software Inc., USA). The classification gradient maps portraying the impact of the studied brands on drug solubility at 24 h as a function of drug aqueous solubility were generated using SigmaPlot 13.0 (Systat Software Inc., USA). For the construction of 3D mesh plots depicting the impact of excipients on drug solubility at 24 h as a function of time and drug ionization, solubility data for PRC, SMX, IBU, DPL were smoothed via the negative exponential technique to allow better visualization using SigmaPlot 13.0 (Systat Software Inc., USA).

In cases where drug intrinsic solubility was not determined experimentally (SMX and DPL in compendial and biorelevant media), the theoretical intrinsic solubility was calculated using the solubility-pH equations (Eqs. 2–5) (48):

2 $$ \log S=\log {S}_o+\log \left({10}^{- pKa+ pH}+1\right)\ \mathrm{for}\ \mathrm{weak}\ \mathrm{acids} $$

3 $$ \log S=\log {S}_o+\log \left({10}^{pKa- pH}+1\right)\ \mathrm{for}\ \mathrm{weak}\ \mathrm{bases} $$

4 $$ \log S=\log {S}_o+\log \left({10}^{+p{Ka}_2+{pKa}_1-2 pH}+{10}^{pKa_2- pH}+1\right)\ \mathrm{for}\ \mathrm{diprotic}\ \mathrm{bases} $$

5 $$ \log S=\log {S}_o+\log \left({10}^{+{pKa}_1- pH}+{10}^{-{pKa}_2+ pH}+1\right)\ \mathrm{for}\ \mathrm{ampholytes} $$

where *S* and *S*_*o*_ indicate the drug solubility at the given pH and the intrinsic solubility, respectively. Deviations in the determination of drug solubility from the aforementioned simplified equations are expected in cases of drug self-association or solubilization in biorelevant media (48). The final pH and experimental solubility values of the ionized drug in basic (for weak acids) or acidic media (for weak bases) were used for the calculation of the theoretical intrinsic solubility. Theoretical pH–solubility profiles in the physiological pH range were constructed to assess if changes in the pH of the medium could justify differences in drug solubility by excipient presence. The final pH and intrinsic solubility values (experimental or theoretical) were used for the construction of the theoretical pH–solubility profiles in the physiological pH range based on Eqs. 2–5.

#### Multivariate Analysis of Solubility Data

Excipient REs on drug apparent solubility were correlated to drug physicochemical properties (drug ionization, drug lipophilicity, drug aqueous solubility), excipient critical material attributes (viscosity, level) and medium characteristics (gastric, intestinal) by partial least squares (PLS) regression using the XLSTAT software (Microsoft, USA). Two models for the REs of excipients on drug apparent solubility in compendial media (model 1) and biorelevant media (model 2) were constructed. The evaluated variables for both models were categorized according to their type as categorical (expressing a category or type) and numerical (measurements with numerical meaning). Categorical variables included (i) drug solubility (low, high), (ii) aminic group (absence, presence), (iii) excipient level (low, high), (iv) medium (gastric, intestinal) while numerical parameters included (i) theoretical % of drug ionized (F_ion_; calculated based on the Henderson–Hasselbalch equation at the pH of each medium), (ii) drug lipophilicity (log P), (iii) excipient viscosity (cP). Excipient REs on drug solubility at 24 h were used as the response. The selected interaction terms included each excipient property combined with each drug physicochemical property (drug ionization, drug lipophilicity, drug aqueous solubility) and medium characteristics (gastric, intestinal). Observation diagnostics were performed prior to model analysis to identify outliers in the data set. The distance of each observation to the model in the Y-plane (DmodY) tool based on PLS residuals was used. Plots of standardized DmodY vs each observation were generated and any observation exceeding the maximum tolerance volume in Y (D_crit(Y)_) was considered an outlier (49,50). Exclusion of outliers was based on (i) deviating cases (positive REs) in solubility caused by a shift in the pH of the solution, (ii) observations resulting in high variability (coefficient of variation (CV%) > 20%) within the triplicate samples (one value from the triplicate could be excluded as the outlier analysis could detect these values). PLS models generated with and without outlier exclusion (data not shown) confirmed that outlier exclusion did not alter the interpretation of results but only enhanced the predictive ability of the regression model. The generated models were assessed in terms of goodness of fit (*R*^2^) and goodness of prediction (*Q*^2^). High values of *R*^2^ and *Q*^2^ with a difference not greater than 0.2–0.3 were indications of successful models (51). The number of PLS components (lines on the X-space which best approximate and correlate with the Y-vector) was based on minimum predictive residual sum of squares (PRESS) (51). From the available components, the one at which *Q*^2^ reached its maximum value was selected (49). Standardized coefficients were used to show the direction (positive or negative) and extent of each variable on the response. The significance of the variables was assessed by the variable influence on projection (VIP) value. VIP values > 0.8 were considered as moderately influential in the model while VIP values > 1 were considered the most influential in the model (51). A 95% confidence interval was used.

#### Roadmap Design

The biopharmaceutical implications of HPMC variability on drug apparent solubility were depicted with the use of roadmap designs. These were constructed by combining the impact of excipients on drug solubility at 24 h from the solubility studies to excipient (viscosity) and drug (drug ionization, drug lipophilicity, drug aqueous solubility) physicochemical properties. Drug categorization was based on drug aqueous solubility and drug lipophilicity (Table I) and drug ionization (low ionized: F_(ion)_ < 50%, highly ionized: F_(ion)_ > 50%). Reference range criteria of − 20% to 25% (47) on the REs of HPMC on drug solubility were set for the risk assessment of the excipient effects on drug solubility. REs of excipients on drug apparent solubility outside these values (REs < − 20% or REs > 25%) were considered to be potentially significant for oral drug performance.

## RESULTS AND DISCUSSION

### Properties of HPMC

Based on product specifications and certificates of analysis, differences in terms of viscosity (cP) for the studied HPMC brands were identified. Pharmacoat 606 (6 cP) (52) and Pharmacoat 615 (15 cP) (52) are low viscosity brands compared to HPMC-Sigma (3500–5600 cP) (53). The studied polymers belong to the same category of substitution type 2910 (28–30% of methoxy and 7–12% hydroxypropoxyl content).

### Solubility Studies

#### Impact of the Studied HPMC Brands on Drug Apparent Solubility

**Fig. 2:**
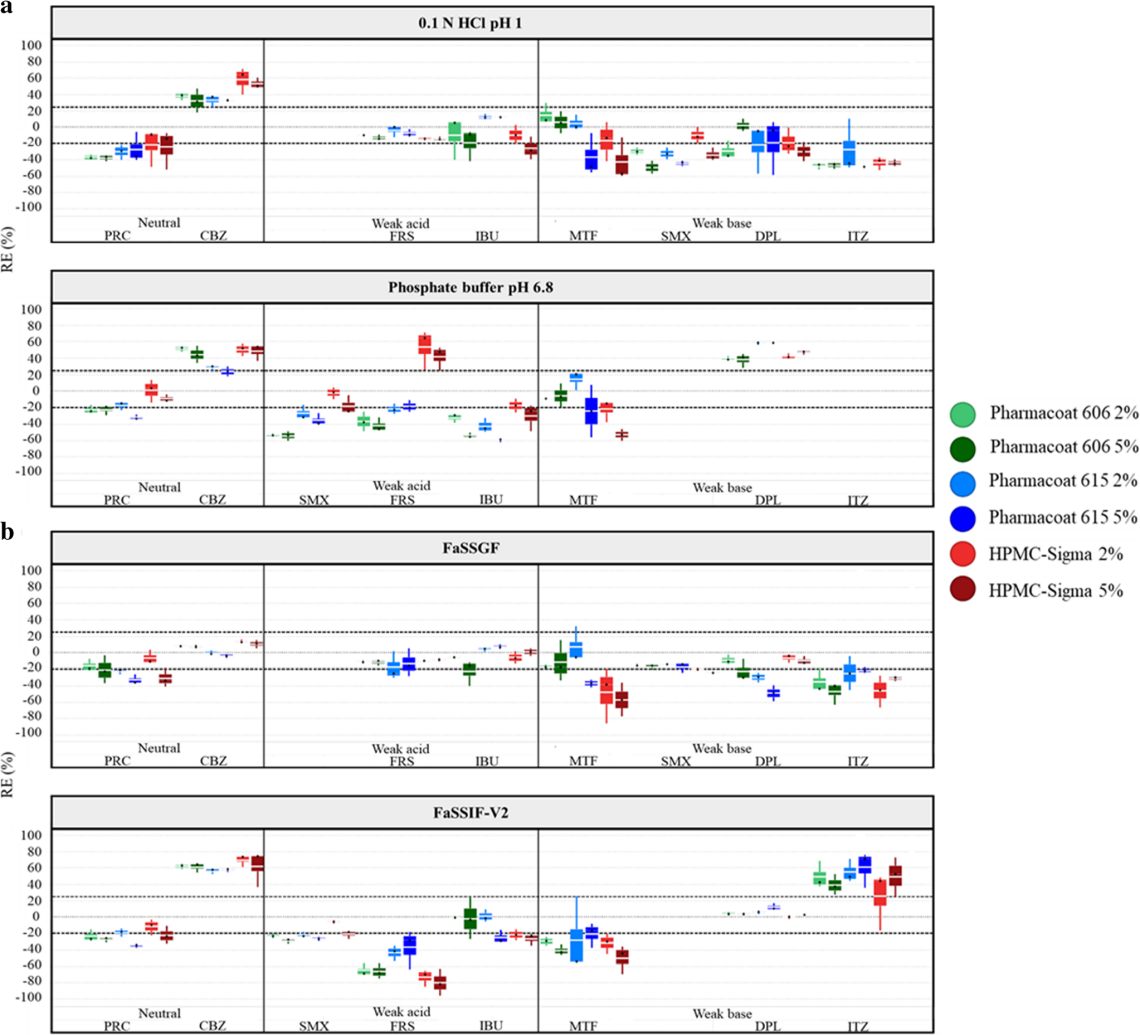
Box plots of the relative effects (%) of the HPMC brands on drug solubility at 24 h in **a** compendial and **b** biorelevant media. The excipient brands are shown as: (i) Pharmacoat 606 (green colour), (ii) Pharmacoat 615 (blue colour) and (iii) HPMC-Sigma (red colour). Light and dark colours correspond to low and high excipient level, respectively. Mean (white line), median (black diamond), *n* = 3

The reference solubility values at 24 h of the studied compounds in compendial and biorelevant media are summarized in Table II. For neutral drugs, the reference solubility did not depend on the pH of the studied media (as expected) but on the presence of solubilizing components, as increased reference solubilities were observed in biorelevant compared to compendial media due to the presence of bile salts (54). Different reference solubility values within acidic and basic media were observed for weak acids (FRS, IBU) and weak bases (DPL, ITZ) due to their ionization pattern, except from the case of MTF as drug was fully ionized in all the studied conditions (34). SMX (weak base and weak acid in acidic and basic media, respectively) was ionized in both acidic and basic media due to its two pKa constants. For weak acids in acidic media (drugs in the unionized state), slight differences in drug solubility were observed between compendial and biorelevant media (IBU: 43 μg/mL in 0.1 N HCl pH 1 vs 44 μg/mL in FaSSGF, FRS: 14 μg/mL in 0.1 N HCl pH 1 vs 15 μg/mL in FaSSGF). For weak bases, drug solubility values in the low ionization drug state were higher in biorelevant as compared to compendial media (DPL: 5 μg/mL in phosphate buffer pH 6.8 vs 13 μg/mL in FaSSIF-V2) which is explained by the presence of bile salts (54) or the higher % of drug ionized in FaSSIF-V2 (34% in FaSSIF-V2 vs 20.1% in phosphate buffer pH 6.8). For weak acids/weak bases in media where drugs are highly ionized, drug solubility values were higher in compendial (0.1 N HCl pH 1, phosphate buffer pH 6.8) compared to biorelevant media (FaSSGF pH 1.6, FaSSIF-V2 pH 6.5), as pH has a greater impact on drug solubility compared to bile salts (55). The effects of the HPMC brands on the solubility of the studied compounds in compendial and biorelevant media are presented in Fig. 2.

**Table II:** Reference Solubility Values (μg/mL) of the Studied Drugs at 24 h in Compendial and Biorelevant Media (mean ± SD, *n* = 3)

	Compendial media		Biorelevant media	
Drug	0.1 N HCl pH 1	Phosphate buffer pH 6.8	FaSSGF	FaSSIF-V2
MTF	3.1 × 10^5^ (± 0.3 × 10^5^)	3.1 × 10^5^ (± 0.2 × 10^5^)	3.4 × 10^5^ (± 0.8 × 10^5^)	4.3 × 10^5^ (± 0.4 × 10^5^)
PRC	1.6 × 10^4^ (± 0.1 × 10^4^)	1.5 × 10^4^ (± 0.1 × 10^4^)	1.7 × 10^4^ (± 0.2 × 10^4^)	1.7 × 10^4^ (± 0.1 × 10^4^)
SMX	1.6 × 10^3^ (± 0.1 × 10^3^)	3.7 × 10^3^ (± 0.1 × 10^3^)	862 (± 21)	1.3 × 10^3^ (± 0.1 × 10^3^)
FRS	14 (± 2)	3.4 × 10^3^ (± 1.4 × 10^2^)	15 (± 1)	1.6 × 10^3^ (± 3.0 × 10^2^)
CBZ	265 (± 6)	227 (± 9)	368 (± 1)	280 (± 7)
DPL	1.3 × 10^4^ (± 9.1 × 10^2^)	5 (± 1)	8.6 × 10^3^ (± 2.0 × 10^2^)	13 (± 1)
IBU	43 (± 3)	5.5 × 10^3^ (± 6.7 × 10^2^)	44 (± 5)	1.5 × 10^3^ (± 5.8)
ITZ	11 (± 1)	–^a^	1.2 (± 0.2)	0.05 (± 0.01)

^a^Below limit of detection of the analytical method

*MTF* metformin, *PRC* paracetamol, *SMX* sulfamethoxazole, *FRS* furosemide, *CBZ* carbamazepine, *DPL* dipyridamole, *IBU* ibuprofen, *ITZ* itraconazole

### Neutral Drugs

**Fig. 3:**
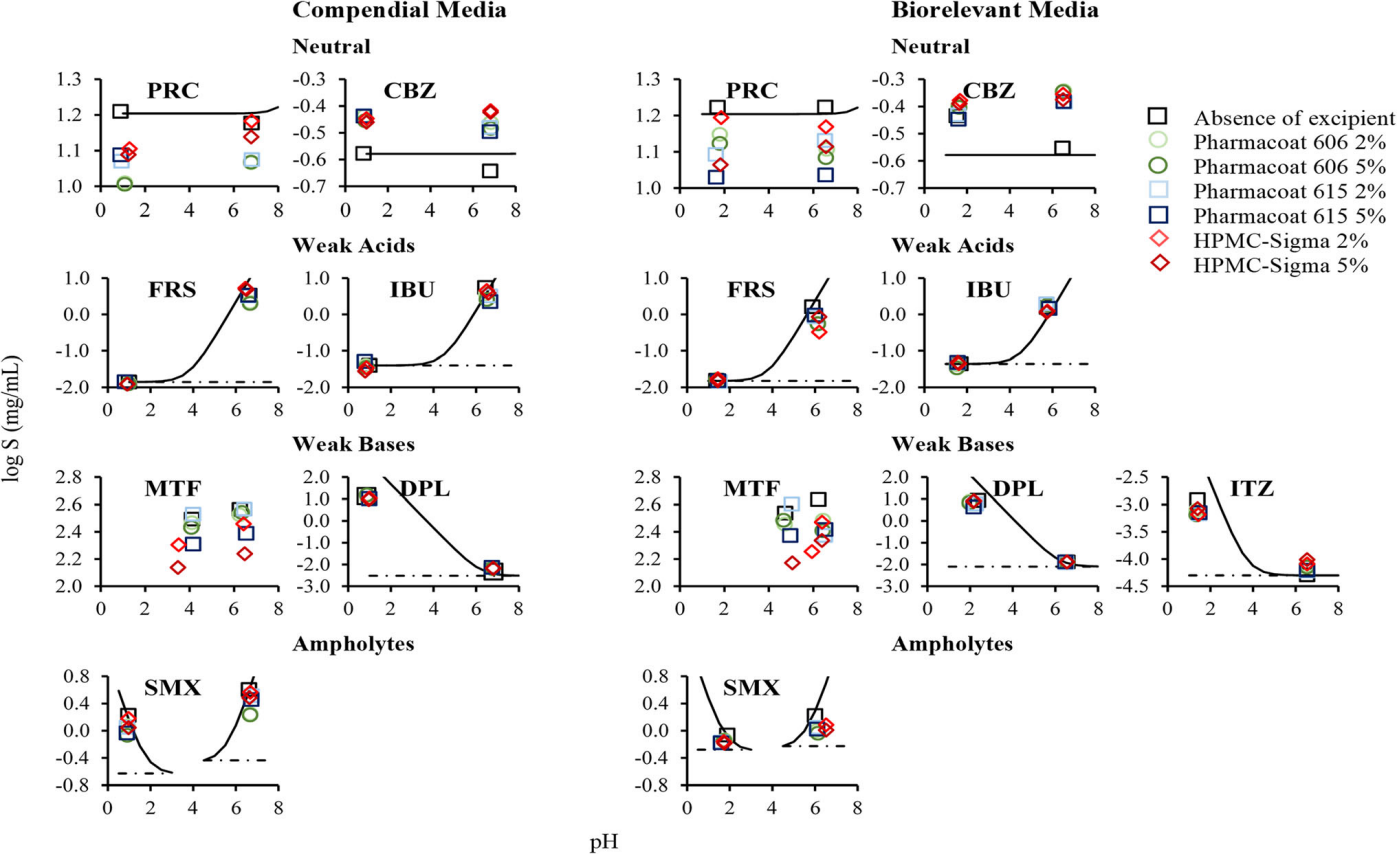
Theoretical pH–solubility profiles of the studied drugs in compendial and biorelevant media and experimental drug solubility values in absence (black colour) and presence of excipients ((i) Pharmacoat 606 (green colour), (ii) Pharmacoat 615 (blue colour), (iii) HPMC-Sigma (red colour)). Dashed lines indicate drug intrinsic solubility

**Fig. 4:**
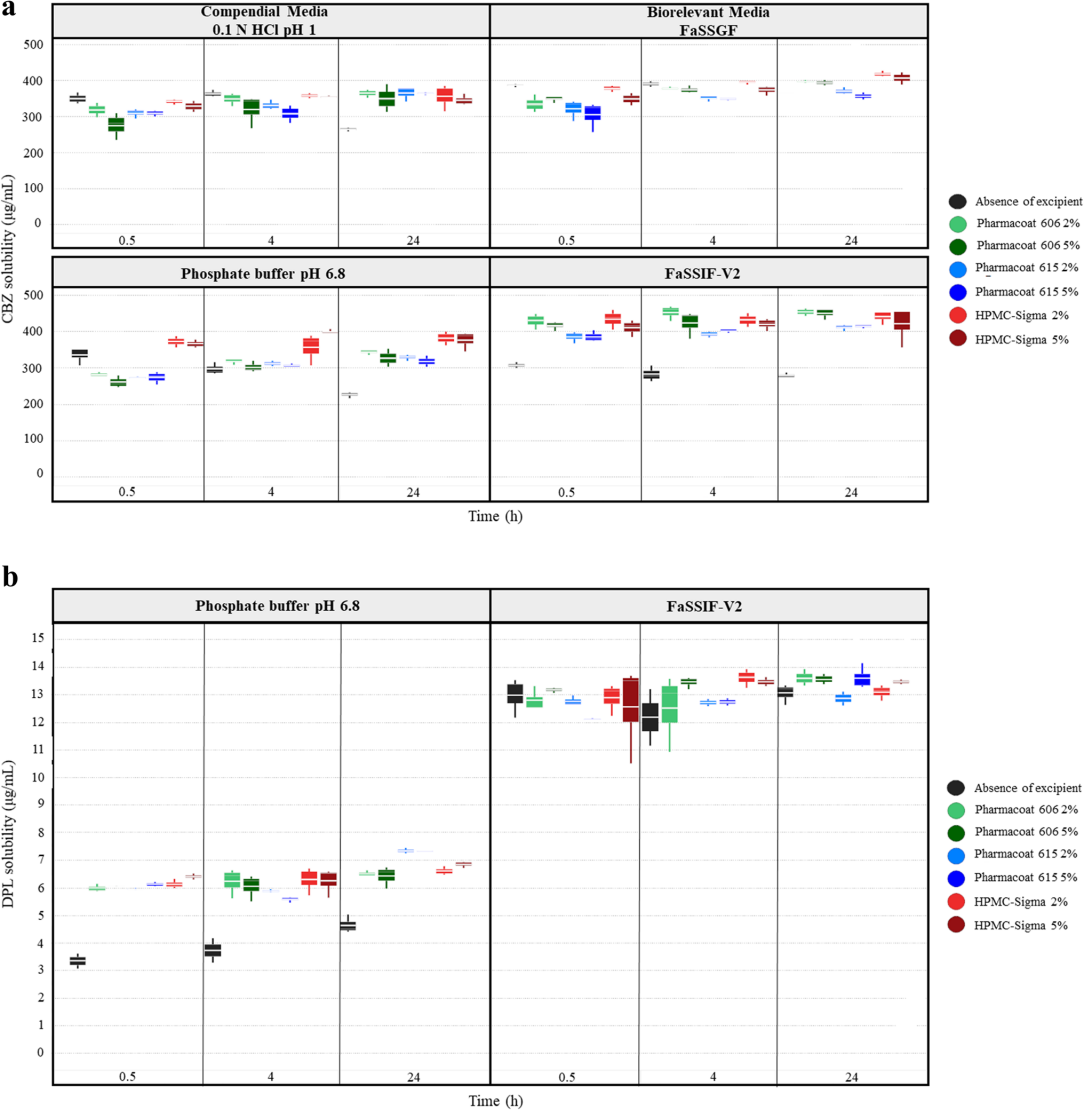
Box plots of **a** CBZ solubility (μg/mL) and **b** DPL solubility (μg/mL) in absence (black colour) and presence of the studied HPMC brands ((i) Pharmacoat 606 (green colour), (ii) Pharmacoat 615 (blue colour) and (iii) HPMC-Sigma (red colour)) in compendial and biorelevant media. Light and dark colours correspond to low and high excipient level, respectively (mean, *n* = 3)

Cases of significant decrease in drug solubility at 24 h by the studied HPMC brands were observed for PRC (Pharmacoat 606: − 36% < REs < − 20%, Pharmacoat 615: − 32% < REs < − 22%, HPMC-Sigma: − 30% < REs < − 22%). Differences in the apparent solubility of neutral drugs in HPMC presence cannot be attributed to changes in the pH of the media (Fig. 3). The pronounced reduction in drug solubility at 24 h by HPMC is attributed to the slow drug dissolution and/or drug solubilization as excipients with slow dissolution rate may shield drug particles from the dissolution medium (56) or the formation of a viscous excipient layer on the powder surface (57). The aforementioned mechanisms indicate that the HPMC will likely affect the kinetic processes of drug dissolution rather than affecting the true thermodynamic drug solubility. Significant increase in drug apparent solubility by the studied HPMC brands was observed for CBZ in compendial media (Pharmacoat 606: 32% < REs < 50%, Pharmacoat 615: 28% < REs < 33%, HPMC-Sigma: 50% < REs < 60%) and in FaSSIF-V2 (REs of approximately 60% for all the studied brands). Solubility data of CBZ at 0.5, 4 and 24 h in absence and presence of the studied HPMC brands in compendial and biorelevant media are presented in Fig. 4a. The solubility of pure CBZ decreased through time in compendial media (350 μg/mL and 250 μg/mL at 0.5 h and 24 h, respectively), potentially due to drug precipitation (58) or due to the solution-mediated phase transformation of CBZ from the anhydrate to the dihydrate form (59). The decrease in CBZ apparent solubility was slower in biorelevant media (FaSSGF: 387 μg/mL to 367 μg/mL at 0.5 h and 24 h, respectively, FaSSIF-V2: 307 μg/mL to 280 μg/mL at 0.5 h and 24 h, respectively). Reduction in CBZ apparent solubility is not observed in presence of HPMC, as potentially dissolved polymer particles may enhance drug solubilization (60). Inhibition of the CBZ solution-mediated phase transformation of by hydrogen bonding (61) or by excipient adsorption in the formed nuclei/crystals delaying the rate of nucleation and crystal growth (59) could also explain the fact that CBZ apparent solubility was not reduced in excipient presence.

### Weak Acids

Cases of significant reduction are observed in weak acidic compound solubility at 24 h, especially in basic conditions (Pharmacoat 606: − 65% < REs < − 20%, Pharmacoat 615: − 40% < REs < − 20%, HPMC-Sigma: − 80% < REs < − 20%) (Fig. 2). In the cases of significant reduction in drug apparent solubility by HPMC presence, small changes in the pH of the media occurred in absence or presence of excipient (± 0.1 pH units) in acidic conditions while the pH of the media in absence or presence of excipient decreased (0.2–0.7 pH units) in basic conditions (potentially due to the dissociation of weak acids). The experimental solubility values in excipient presence were lower compared to theoretical ones (expected by the change in the pH of the media) (Fig. 3). As the pronounced reduction in drug solubility at 24 h by HPMC cannot be explained by the shift in the pH of the media, it is attributed to the delay in drug dissolution and/or drug solubilization, as explained previously in the case of neutral drugs. Significant increase in drug apparent solubility was only observed for FRS in presence of both levels of HPMC-Sigma in phosphate buffer pH 6.8 (REs of 53% and 41% for the low and high level, respectively). Differences in the pH of the medium in presence (reduction of 0.3 pH units) and absence of excipient (reduction of 0.2 pH units) were observed. Evaluation of the theoretical pH–solubility profile (Fig. 3) reveals that drug solubility in absence of excipient does not correspond to the theoretical drug solubility (expected according to the change in the pH of the medium). In presence of HPMC-Sigma, the experimental and theoretical solubility values are similar, therefore the increase in drug solubility at 24 h in presence of HPMC-Sigma is attributed to the change of the pH and not to a potential drug-excipient interaction (further investigations are needed to explain the nature of this change, as shifts in the pH of the media by the non-ionic HPMC are not expected).

### Weak Bases

For the majority of cases, significant reduction is observed in weak basic compound solubility at 24 h by HPMC presence (− 50% < REs < − 20% for all the studied HPMC brands) (Fig. 2). The impact of pH on the solubility of MTF, DPL and ITZ cannot be evaluated due to *in situ* salt formation between the drugs and counterions of the medium (62) (Fig. 3). For SMX in acidic media, the changes in the pH of the media observed in presence of HPMC were small (± 0.1 pH units). The differences in SMX solubility in HPMC presence cannot be attributed to the pH change of the media as the experimental solubility values in excipient presence were lower compared to theoretical ones (Fig. 3). As per neutral drugs and weak acids, presence of HPMC affected the kinetic processes of drug dissolution/solubilization leading to reduced drug apparent (rather than equilibrium) solubility. Significant increase in drug solubility at 24 h was observed for DPL in phosphate buffer pH 6.8 (38% < REs < 58%) and ITZ in FaSSIF-V2 (25% < REs < 61%) in presence of all the studied HPMC brands. Solubility data of DPL at 0.5, 4 and 24 h in absence and presence of HPMC in phosphate buffer pH 6.8 and FaSSIF-V2 are presented in Fig. 4b. In phosphate buffer pH 6.8, increased DPL apparent solubility in presence of HPMC is observed even at early time points (0.5 and 4 h) and can be attributed to intermolecular interactions between the amine group of DPL and the hydroxyl group of HPMC which improve drug solubilization (63). Presence of HPMC did not affect the DPL apparent solubility in FaSSIF-V2 potentially due to the enhanced drug solubilization by the bile salts. ITZ forms a supersaturated solution in FaSSIF-V2 due to the micellar solubilization effect of bile salts and slowly precipitates with time (64). The increase in ITZ apparent solubility in HPMC presence in FaSSIF-V2 can be attributed to the inhibition of drug precipitation by HPMC due to potential interaction of the neutral aminic group of ITZ with the hydroxyl groups of HPMC.

The solubility data showed increased variability in the cases where HPMC presence significantly affected drug solubility (MTF: CV% > 40%, PRC or highly ionized poorly soluble drugs: 10% < CV% < 30%). As working with physical mixtures may yield high standard deviations due to the heterogeneous dispersion of the constituents (65,66), the increased variability can be attributed to the heterogeneous saturation of powder surface with excipient particles.

### Impact of Excipients on Drug Apparent Solubility Based on Drug Physicochemical Properties

**Fig. 5:**
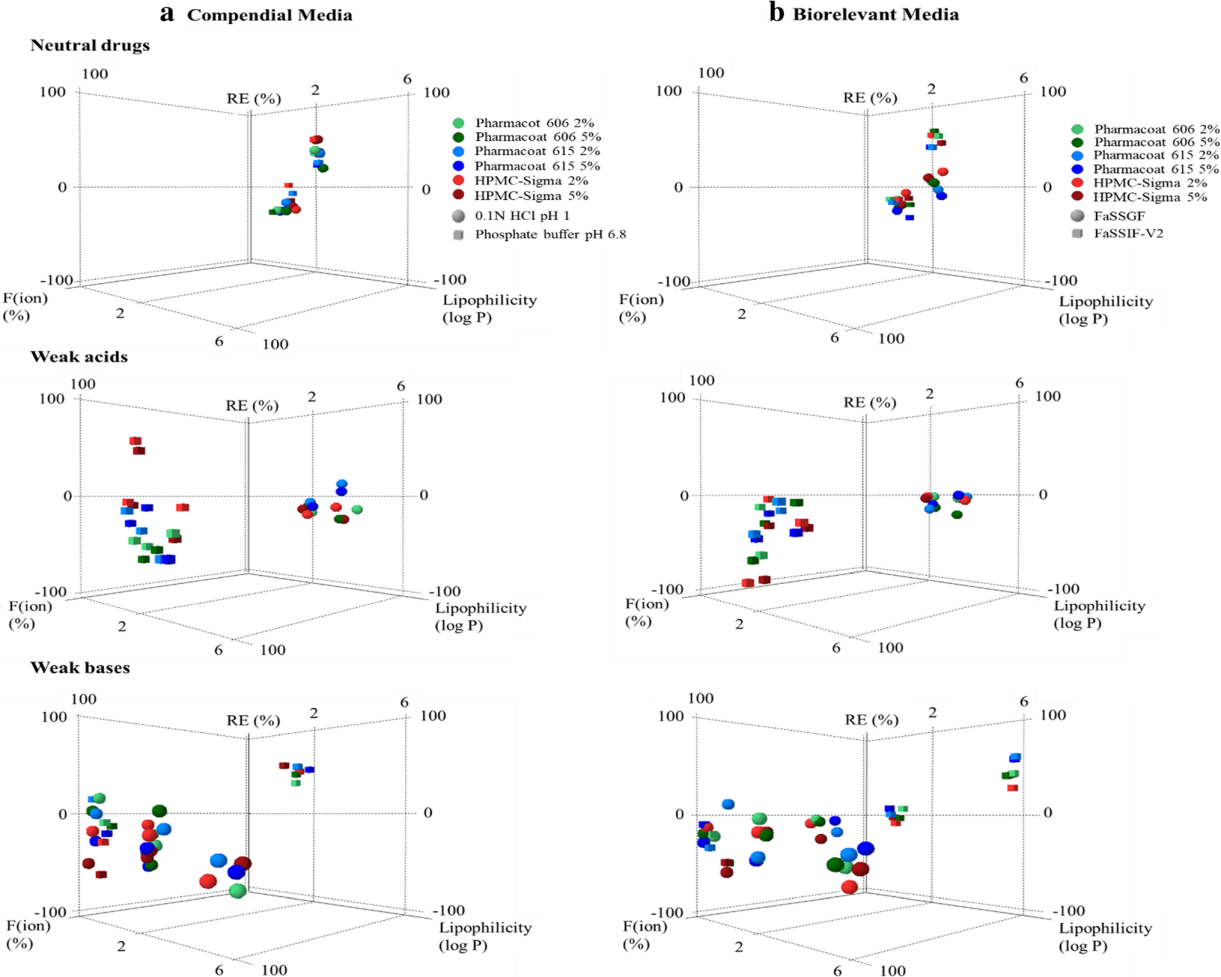
Relative effects (%) of the studied HPMC brands on drug solubility at 24 h as a function of drug ionization (%) and drug lipophilicity (log P) in **a** compendial and **b** biorelevant media. The excipient brands are shown as: (i) Pharmacoat 606 (green colour), (ii) Pharmacoat 615 (blue colour), (iii) HPMC-Sigma (red colour). Light and dark colours correspond to low and high excipient levels, respectively. Media representing gastric and intestinal conditions are presented as (i) acidic media (circles) and (ii) basic media (squares)

**Fig. 6:**
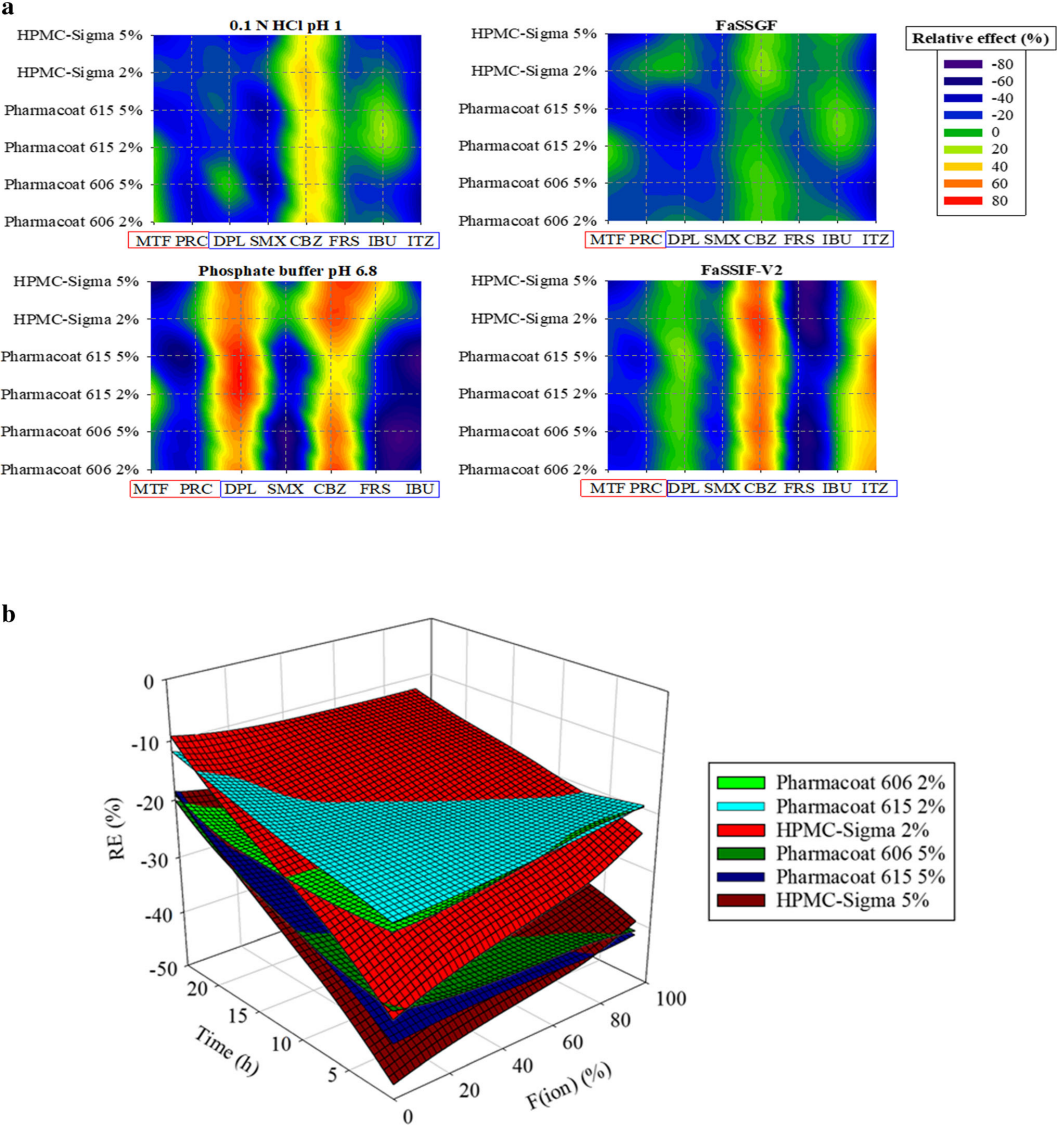
**a** Classification gradient maps of the relative effects of the HPMC brands on the solubility of highly and poorly soluble compounds at 24 h. Y-axes are set in an increasing excipient viscosity type and excipient level order. The x-axes are set in a decreasing drug aqueous solubility order (red colours for highly soluble and blue colours for poorly soluble drugs). **b** Relative effects of (i) Pharmacoat 606 (green colour), (ii) Pharmacoat 615 (blue colour) and (iii) HPMC-Sigma (red colour) on drug solubility as a function of drug ionization (%) and time (h). Light and dark colours correspond to low and high excipient level, respectively

The effects of the studied HPMC brands on the apparent solubility of neutral drugs, weak acids and weak bases as a function of drug ionization and drug lipophilicity in compendial and biorelevant media are presented in Fig. 5. For neutral drugs, the different effects of the HPMC brands on drug solubility at 24 h (decrease and increase for the solubility values of PRC and CBZ, respectively) are attributed to the differences in drug lipophilicity between the two compounds (Table I). For weak acids and weak bases, significant decrease in drug apparent solubility was observed in media (compendial or biorelevant) where drugs are highly ionized (excluding the cases of increased drug solubility attributed to the change of the pH of the medium). A trend between the impact of excipients on the 24-h drug solubility and drug lipophilicity was found for weak bases in biorelevant media where drugs are unionized, as increased drug solubility was observed in HPMC presence for highly lipophilic drugs. The decrease in drug apparent solubility by HPMC presence was more pronounced with increasing level of drug ionization and/or decreasing drug lipophilicity (Fig. 5**)** which can be explained by the presence of an increased number of excipient molecules on the powder surface (56,67). Reduced drug mobility and slower drug diffusion have been reported for charged molecules due to interactions with the polymeric chains of HPMC (57) and may also have contributed to the decrease in the apparent solubility of ionized molecules by HPMC. The classification gradient map depicting the effects of the studied HPMC brands on drug solubility at 24 h as a function of decreasing drug aqueous solubility in compendial and biorelevant media is presented in Fig. 6a. The map confirms that the decrease in drug apparent solubility in presence of HPMC was more pronounced for highly soluble drugs which are inherently less lipophilic while for poorly soluble drugs, the reduction in drug solubility at 24 h was more pronounced in media where drugs were highly ionized.

#### Impact of Excipients on Drug Solubility Based on Excipient Properties

The effects of the studied HPMC brands on drug apparent solubility as a function of drug ionization and time for drugs were solubility data at early time points were available (PRC, SMX, CBZ, DPL and IBU) are presented in Fig. 6b. Solubility data of CBZ in compendial and biorelevant media and of DPL in phosphate buffer pH 6.8 were not included in this visualization, as significant increase in drug solubility was observed. The decrease in drug apparent solubility by HPMC presence was more pronounced at early time points. When HPMC is used at low levels (< 10% *w*/*w*) in tablet formulations, a heterogeneous viscous HPMC layer is formed initially and cannot be maintained due to water penetration (19). Therefore, the higher reduction in drug apparent solubility at early compared to late time points may be explained by the disruption of the gel layer by water molecules. The high viscosity brand (HPMC-Sigma) resulted in higher reduction in drug apparent solubility at early time points compared to the low viscosity brands (Pharmacoat 606, Pharmacoat 615) which can be explained by the formation of a stronger gel layer by the high HPMC viscosity types compared to the low ones (9,13,14). The time-dependent impact of HPMC on drug solubility reveals that the excipient affects the kinetic processes of drug dissolution and mostly influences the drug apparent rather than the true equilibrium drug solubility. Increasing the level of all the studied HPMC brands resulted in higher reduction in drug apparent solubility compared to the low excipient level, as the increased number of chain entanglements, when increasing excipient level, leads to the formation of strong gel layer (16) which further delays drug dissolution and/or drug solubilization.

### Multivariate Data Analysis

**Fig. 7:**
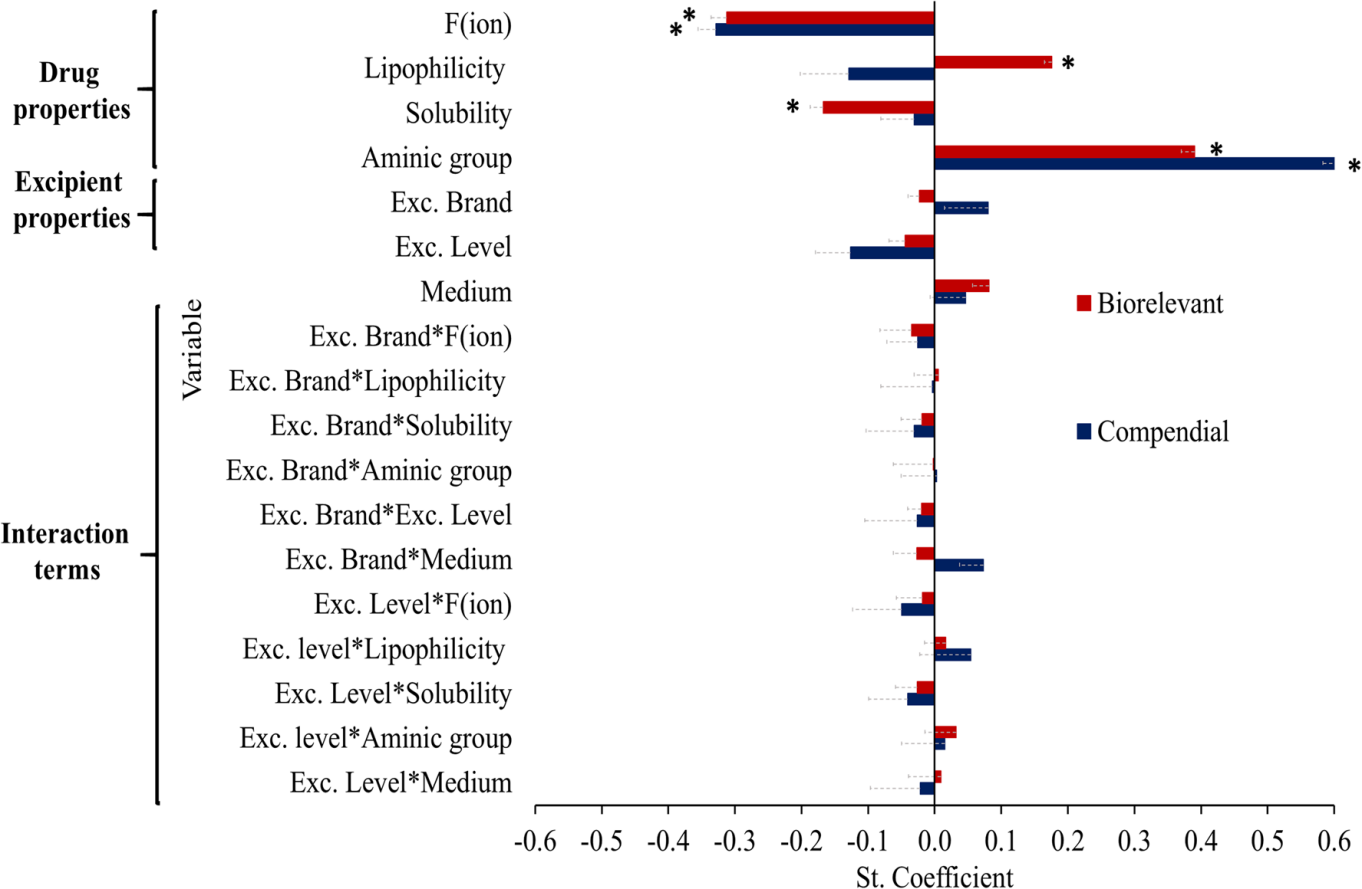
Standardized coefficients of the studied variables (and interaction terms) in compendial (blue colour) and biorelevant (red colour) media. Asterisk denotes coefficients of VIP > 1 (mean, −SE)

The standardized coefficients of the variables in compendial and biorelevant media are presented in Fig. 7. The two models showed a good predictive power and fit (compendial media: 2 principal components, *Q*^2^ = 0.7 and *R*^2^ = 0.7, biorelevant media: 1 principal component, *Q*^2^ = 0.6 and *R*^2^ = 0.6). The statistical analysis reveals that the impact of HPMC on drug apparent solubility strongly depends on the physicochemical properties of the studied brands. Amine group (compendial media: positive effect, VIP = 3.2; biorelevant media: positive effect, VIP = 2.9) was a significant variable in both models. The variable aminic group term indicates that pronounced increase in drug apparent solubility in HPMC presence is expected for drugs containing a neutral aminic group due to a potential drug-HPMC interaction which enhances drug solubilization. Pronounced reduction in drug apparent solubility is anticipated for highly ionized drugs, potentially due to the slower drug mobility and diffusivity by the presence of the viscous HPMC layer (57) as indicated by the significance of drug ionization (compendial media: negative effect, VIP = 2.2; biorelevant media: negative effect, VIP = 2.3) in both models. In biorelevant media, drug lipophilicity (positive effect, VIP = 1.3) and drug solubility (negative effect, VIP = 1.3) were also significant variables in the statistical models. Both variables indicate that pronounced reduction is expected for highly soluble/less lipophilic drugs as the enhanced drug solubilization caused by the presence of bile salts can result in saturation of the powder surface by HPMC particles. The statistical analysis demonstrates that, when HPMC is used as a binder, excipient variability and variation are not significant parameters for the impact of HPMC on drug solubility.

### Road Map of Excipient Effects on Drug Apparent Solubility

**Fig. 8:**
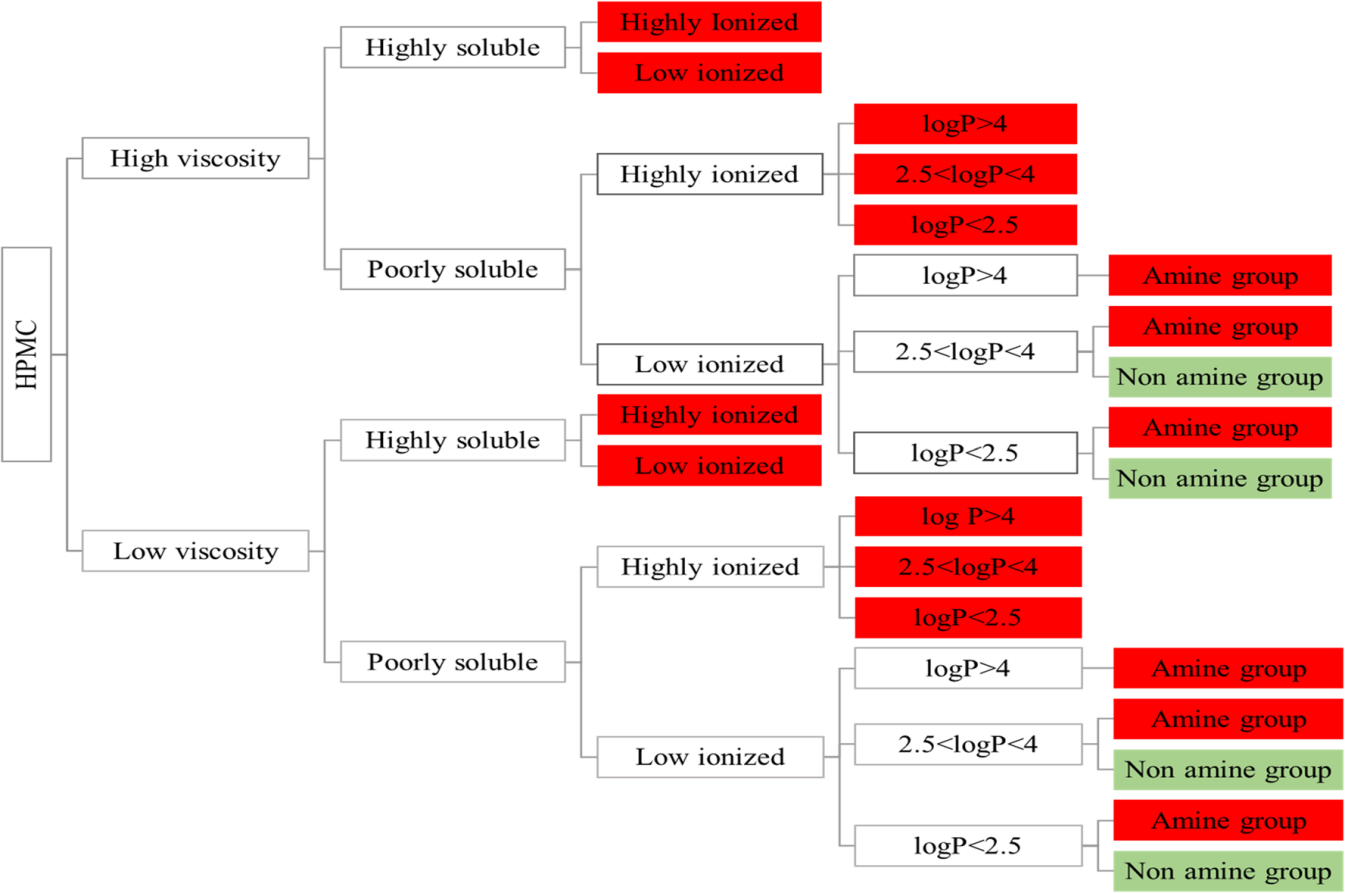
Road map of the effects of the studied excipients (low viscosity: Pharmacoat 606, Pharmacoat 615, high viscosity: HPMC-Sigma) on drug solubility. Red boxes and green boxes indicate significant and insignificant changes in drug solubility by excipient presence, respectively

The road map categorizing the excipient REs on drug apparent solubility according to excipient (HPMC) and drug properties is presented in Fig. 8 (cases where increased drug solubility was caused by a potential shift in the pH of the medium were not considered). The impact of the studied HPMC brands on drug solubility at 24 h depends on the physicochemical properties of the studied compounds. Significant changes (decrease) in drug apparent solubility by HPMC brands of different viscosity type are anticipated for highly soluble drugs, irrespective of drug ionization state (low or highly ionized). For poorly soluble drugs, the impact of the studied HPMC brands on drug apparent solubility depends on the drug ionization state, as significant decrease in drug solubility at 24 h is expected when poorly soluble drugs are highly ionized (irrespective of drug lipophilicity). For poorly soluble/low ionized, HPMC is not found to affect drug apparent solubility, apart from drugs containing a neutral aminic group and for which presence of HPMC may result in significant increase in drug solubility. The generated roadmap reveals that presence of HPMC may be challenging for oral drug performance, as differences in drug apparent solubility caused by the excipient may complicate oral drug absorption and bioavailability.

## CONCLUSIONS

Excipient presence, variability and variation can affect product performance and present challenges for oral drug bioavailability. HPMC is a commonly used binder in immediate release formulations but can compromise drug dissolution and/or drug solubilization as it forms a viscous layer around particles. In this work, the impact of HPMC viscosity type on drug apparent solubility was assessed in a biopharmaceutical perspective. Solubility studies showed that presence of the studied HPMC brands can significantly change drug apparent solubility, but its magnitude depends on drug physicochemical properties. Significant reduction in the 24-h solubility of highly soluble or highly ionized drugs was observed potentially due to the formation of a viscous layer by HPMC. For poorly soluble drugs containing a neutral aminic group, increase in drug apparent solubility was observed in HPMC presence attributed to the enhanced drug solubilization by the polymeric HPMC chains. Increasing HPMC viscosity and/or level resulted in pronounced decrease in drug apparent solubility especially at early time points, as at the studied excipient levels, the formation of a viscous layer is disrupted by water penetration. The use of multivariate data analysis and the construction of roadmaps revealed that the effects of HPMC on solid oral dosage form performance needs to be studied to better understand the potential impact of these effects on product performance. It is concluded that HPMC presence and variability may present challenges for oral drug absorption and that expanding current knowledge to other excipient types would be beneficial for the successful implementation of excipient variability on QbD approaches.

## Electronic supplementary material

ESM 1 (DOCX 17 kb)
